# Metallothioneins 1 and 2 Modulate Inflammation and Support Remodeling in Ischemic Cardiomyopathy in Mice

**DOI:** 10.1155/2016/7174127

**Published:** 2016-06-14

**Authors:** Georg D. Duerr, Daniela Dewald, Eva J. Schmitz, Luise Verfuerth, Katharina Keppel, Christine Peigney, Alexander Ghanem, Armin Welz, Oliver Dewald

**Affiliations:** ^1^Department of Cardiac Surgery, University Clinical Center, Rheinische Friedrich-Wilhelms-Universität Bonn, Sigmund-Freud-Straße 25, 53127 Bonn, Germany; ^2^Department of Anesthesiology, University Clinical Center, Rheinische Friedrich-Wilhelms-Universität Bonn, Sigmund-Freud-Straße 25, 53127 Bonn, Germany; ^3^Department of Ophthalmology, University Clinical Center, Rheinische Friedrich-Wilhelms-Universität Bonn, Sigmund-Freud-Straße 25, 53127 Bonn, Germany; ^4^Department of General, Visceral, Thoracic and Vascular Surgery, University Clinical Center, Rheinische Friedrich-Wilhelms-Universität Bonn, Sigmund-Freud-Straße 25, 53127 Bonn, Germany; ^5^Department of Medicine II-Cardiology, University Clinical Center, Rheinische Friedrich-Wilhelms-Universität Bonn, Sigmund-Freud-Straße 25, 53127 Bonn, Germany

## Abstract

*Aims*. Repetitive brief ischemia and reperfusion (I/R) is associated with left ventricular dysfunction during development of ischemic cardiomyopathy. We investigated the role of zinc-donor proteins metallothionein MT1 and MT2 in a closed-chest murine model of I/R.* Methods*. Daily 15-minute LAD-occlusion was performed for 1, 3, and 7 days in SV129 (WT)- and MT1/2 knockout (MT^−/−^)-mice (*n* = 8–10/group). Hearts were examined with M-mode echocardiography and processed for histological and mRNA studies.* Results*. Expression of MT1/2 mRNA was transiently induced during repetitive I/R in WT-mice, accompanied by a transient inflammation, leading to interstitial fibrosis with left ventricular dysfunction without infarction. In contrast, MT^−/−^-hearts presented with enhanced apoptosis and small infarctions leading to impaired global and regional pump function. Molecular analysis revealed maladaptation of myosin heavy chain isoforms and antioxidative enzymes in MT1/2^−/−^-hearts. Despite their postponed chemokine induction we found a higher total neutrophil density and macrophage infiltration in small infarctions in MT^−/−^-hearts. Subsequently, higher expression of osteopontin 1 and tenascin C was associated with increased myofibroblast density resulting in predominately nonreversible fibrosis and adverse remodeling in MT1/2^−/−^-hearts.* Conclusion*. Cardioprotective effects of MT1/2 seem to be exerted via modulation of contractile elements, antioxidative enzymes, inflammatory response, and myocardial remodeling.

## 1. Introduction

The concept of brief repetitive episodes of myocardial ischemia and reperfusion (I/R) has been introduced as an important mechanism during development of ischemic heart disease [1]. Brief repetitive I/R episodes are not sufficient to induce myocardial infarction but are potent enough to trigger functional and morphological changes in the human heart resulting in a state of myocardial hibernation. This condition aims to preserve the myocardial integrity under conditions of impaired blood supply, that is, stenosis in coronary artery disease, at the cost of temporarily impaired myocardial function [2]. Upon restoration of a normal blood flow after revascularization therapy, the myocardial function recovers within a six-month period [1]. This restoration of function has been associated with the presence of newly recruited leukocytes and low level of inflammation in myocardial segments with restoration of function. Based on these clinical findings we developed a murine model of brief repetitive I/R [3]. This model has a few characteristics comparable to the human hibernating myocardium: transient inflammatory reaction, reversible interstitial fibrosis, and reversible left ventricular dysfunction. We identified the presence of reactive oxygen species [3] and of the chemokine CCL2 [4] to be important for development of interstitial fibrosis without a loss of cardiomyocytes. In following studies we were able to depict mechanisms of cardioprotection and found an irreversible loss of cardiomyocytes and deteriorated cardiac function in mice deficient in endocannabinoid receptor CB2 [5] and in matricellular glycoprotein osteopontin 1 [6]. In both studies, the cardiomyocyte loss was associated with a significantly lower expression of metalloprotein MT1 and MT2, in contrast to their persistent induction in the WT-mice.

Induction of MT, a highly conserved zinc-storage protein, has been reported under several stress conditions involving the mechanisms of NO-mediated release of zinc [7] or inflammatory diseases [8]. Several studies utilized mice with cardiac-specific overexpression of metallothionein. One of them described attenuation of I/R injury in these mice [9], while another reports antiapoptotic effects after I/R [10]. A recent study provided evidence for attenuated myocardial remodeling in MT-overexpressing mice [11]. Few studies investigated the underlying mechanisms and reported association of MT with STAT3-mediated cardioprotection [12] and induction of Akt-pathway in prevention of cell death [13]. Primary culture of cardiomyocytes from mice deficient in MT1 and MT2 experienced increased toxicity and ROS generation after doxorubicin treatment [14]. Based on these results and our previous work, we postulated an important role for MT1/2 in cardioprotection and investigated the underlying mechanisms in MT1-/MT2-deficient mice during repetitive I/R.

## 2. Methods

### 2.1. Study Animals

We used transgenic mice with homozygous 129S7/SvEvBrd-*Mt1* ^*tm1Bri*^ *Mt2* ^*tm1Bri*^/J mutation of the metallothionein 1 and 2 genes (MT^−/−^) leading to a complete loss of their function [15]. These mice were obtained from The Jackson Laboratory (Bar Harbor, ME, USA) and compared to their background strain SV129 wildtype (WT)-mice, obtained from Charles River (Sulzfeld, Germany). All experiments were performed in mice of 20–25 g and 10–12 weeks of age. The study was in accordance with an animal protocol approved by the local governmental authorities and according to the EU Directive 2010/63/EU for animal experiments.

### 2.2. Brief, Repetitive I/R Procedure

For occlusion of the left descending coronary artery (LAD), an 8-0 Prolene suture (Ethicon, Norderstedt, Germany) was placed around it in an initial surgery and then stored subcutaneously as previously described [16]. Mice with initial surgery and 7-day recovery period without I/R were used as sham animals. After a 7-day recovery period the skin was reopened and occlusion of the LAD for 15 min with subsequent reperfusion were monitored in simultaneous ECG recording. This procedure was repeated for 1, 3, or 7 days as previously described and the hearts were excised five hours after the last ischemia episode [3]. Echocardiographic evaluation was performed in the 7-day group before heart retrieval. For histological experiments whole hearts were excised, rinsed with ice-cold cardioplegic solution, and fixated in zinc-paraformaldehyde (Z-fix, 4%; Anatech, Battle Creek, MI, USA). For mRNA studies hearts were further dissected free from great vessels and atria and immediately stored in RNA-later (Qiagen, Hilden, Germany) at 4°C [17].

### 2.3. Echocardiography

M-mode echocardiography in the parasternal short axis view at the level of the papillary muscle was performed using HDI-5000 system and a linear 15 MHz transducer (CL15-7; both ATL–Phillips, Oceanside, CA, USA) on anesthetized animals (2% isoflurane for induction and 0.8–1.2% in 100% O_2_ for maintenance of anesthesia) [18]. Global left ventricular function was estimated by calculation of the fractional shortening (FS) and regional function of the left ventricular anterior wall via calculation of the anterior wall thickening (AWT) [19]. In order to estimate anesthesia-related cardiodepressive effects, the heart rate was monitored continuously and compared between both groups.

### 2.4. Basic Histology

Hearts were paraffinized and cut from basis to apex. Sections of 5 *μ*m thickness from the papillary muscles insertion level were stained for basic histopathology (hematoxylin and eosin, HE) as already described [20]. Planimetric quantification of total collagen stained with picrosirius red (SR, Sigma-Aldrich, Steinheim, Germany) was presented as a percentage of the total left ventricular wall area, as already published [20]. Microinfarcted areas were related to the total collagen stained area and presented as a percentage.

### 2.5. Immunohistology

The following primary antibodies were used: alpha smooth muscle actin (*α*-SMAC) mouse monoclonal antibody (clone 1A4; Sigma, St. Louis, MO, USA), macrophages MAC-2 clone 3/38 rat antibody (AXXORA, Lörrach, Germany), neutrophils MCA771G (Ly-6B.2) clone 7/4 rat anti-mouse monoclonal antibody (MorphoSys, Oxford, UK), and tenascin C rabbit anti-chicken polyclonal antibody (Chemicon, Temecula, CA, USA). The following kits were used: M.O.M immunodetection kit for mouse-derived antibodies (AXXORA), Vectastain Elite ABC kits (Vector, Burlingame, CA, USA), and diaminobenzidine (AXXORA). Planimetric analysis of *α*-SMAC^+^ area as percentage to anterior left ventricular wall was performed on two pictures with a magnification of 200x as published [20]. Cell density of macrophages and neutrophils was evaluated by manual cell count on four pictures (magnification 400x) taken from the anterior left ventricular wall [19].

### 2.6. Apoptosis Staining

Nuclei with DNA-fragmentation were stained with TUNEL In Situ Cell Death Detection Kit (POD, Roche, Mannheim, Germany) according to the manufacturer's protocol. The fluorescence signal was converted and then stained with diaminobenzidine (AXXORA) and slides were counterstained with Quick hemalaun kit (Vector) [19].

### 2.7. Gene Expression

The mRNA expression was measured with Taqman real time quantitative PCR system (RT-qPCR, Applied Biosystems, Foster City, CA, USA). Isolation of total RNA was performed with phenol/chloroform extraction (Trizol, Invitrogen). High capacity cDNA transcription kit with random hexameric primers (Applied Biosystems) was used for the synthesis of first-strand cDNA as described in the manufacturer's protocol. Taqman RT-qPCR was performed with 1/10 diluted cDNA on an ABI Prism 7900HT Sequence Detection System and SDS2.4 Software (Applied Biosystems). FAM-TAMRA® and the relative standard curve method were employed for the measurement of all primers. In order to ascertain the amplification of a single PCR product, dissociation curve analysis was performed. The mRNA expression was related to shams and GAPDH using comparative ΔΔCt-method [6, 19].

### 2.8. Statistical Analysis

Normal distribution of the data was tested and data presented as mean ± SEM. One-way ANOVA with Newman-Keuls* post hoc* testing using GraphPad PRISM (Version 5.0, Graphpad Inc., La Jolla, CA, USA) was performed. When comparing two groups, unpaired *t*-test was chosen. Differences with *P* ≤ 0.05 were considered statistically significant.

## 3. Results

### 3.1. Deficiency in MT1 and MT2 Is Associated with Profound Left Ventricular Dysfunction and Cardiomyocyte Loss

**Figure 1:**
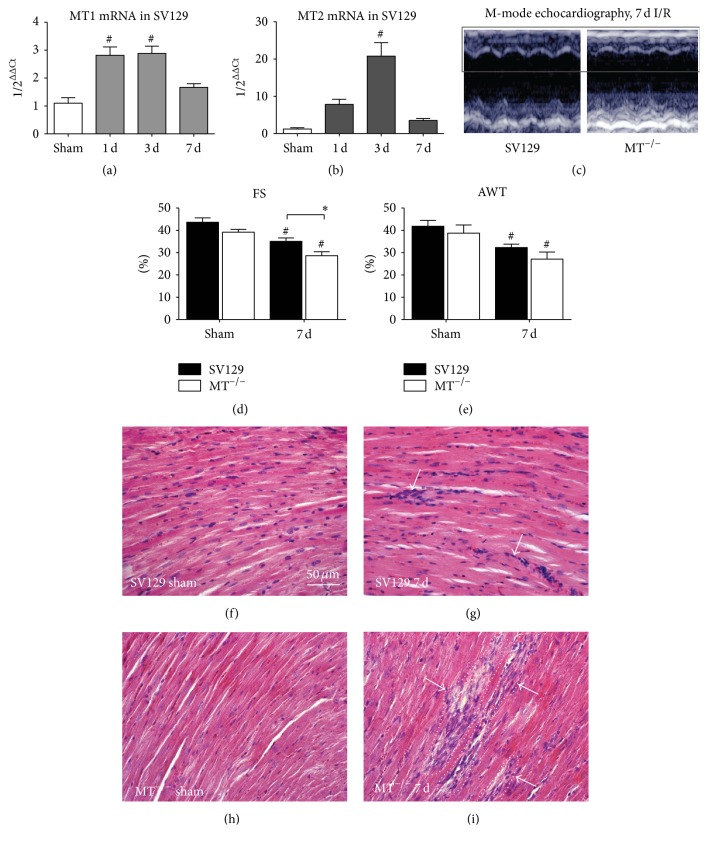
MT1-/2-deficiency leads to cardiomyocyte loss and deterioration of left ventricular function. The mRNA of (a) MT1 and (b) MT2 is upregulated after 1, 3, and 7 days (d) I/R in SV129 WT-mice. (c) Representative short axis M-mode echocardiographs from WT- and MT^−/−^-mice after 7 days of (d) I/R. (d) and (e) reveal worse left ventricular function of MT^−/−^-mice after 7 days of I/R compared to the WT. Representative HE-stainings reveal no cellular infiltration in both sham groups (f and h), but interstitial cellular infiltrates (arrows) into WT-hearts after 7 days of I/R (g), in contrast to microinfarcted areas with loss of cardiomyocytes (dotted line) and cellular infiltration (arrows) in MT1^−/−^-mice at the same time (h and i). *n* = 8–10/group; scale bar in (f)–(i): 50 *μ*m; RT-qPCR using Taqman®, mRNA expression is related to shams and GAPDH using comparative ΔΔCt-method; *∗* indicates* P* ≤ 0.05 between the genotypes; # indicates* P* ≤ 0.05 versus respective sham.

Initially, we measured expression of MT1 and MT2 mRNA in WT-mice and found a transient upregulation of both during repetitive I/R (Figures 1(a) and 1(b)). The M-mode echocardiography measurements revealed a significantly lower fractional shortening and anterior wall thickening in both strains when compared to their respective shams (Figures 1(c)–1(e)). At the same time the MT^−/−^-mice had a significantly worse fractional shortening than the WT-mice. HE staining showed no cellular infiltrations and intact myocardial structure in sham operated mice of both genotypes (Figures 1(f) and 1(h)). After 7 days of repetitive I/R an interstitial cellular infiltration in absence of cardiomyocyte loss was found in WT-hearts (Figure 1(g)) in contrast to MT^−/−^-mice, which presented with cardiomyocyte loss in small, clearly delineated areas of infarction—microinfarctions—after 3 and 7 days of I/R (Figure 1(i)).

### 3.2. Apoptosis and Impaired Cellular Adaptation in MT^−/−^-Mice

**Figure 2:**
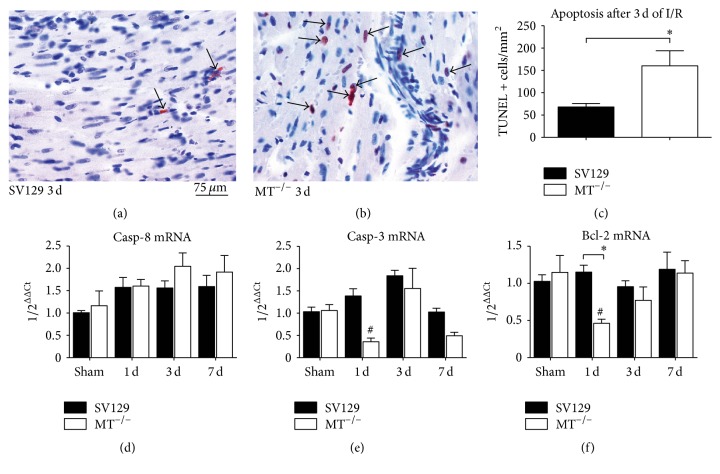
Increased cardiomyocyte apoptosis in MT^−/−^-hearts. Representative slides of TUNEL stained sections present (a) only few apoptotic nuclei in cardiomyocytes of WT-hearts, when compared to (b) numerous nuclei with DNA-fragmentation in MT^−/−^-hearts after 3 days of (d) I/R. (c) TUNEL-positive cells after 3 days of I/R. RT-qPCR showing mRNA expression of (d) caspase- (Casp-) 8, (e) caspase- (Casp-) 3, and (f) B-cell lymphoma (Bcl)-2 during repetitive I/R. *n* = 8–10/group; scale bar in (a) and (b): 75 *μ*m; RT-qPCR using Taqman, mRNA expression is related to shams and GAPDH using comparative ΔΔCt-method; *∗* indicates* P* ≤ 0.05 between the genotypes; # indicates* P* ≤ 0.05 versus respective sham.

In the next step we further investigated the loss of cardiomyocytes using TUNEL staining for apoptosis. WT-mice showed only a few scattered TUNEL-positive nuclei in the ischemic myocardium after 3 days of I/R (Figure 2(a)). In contrast, MT^−/−^-mice presented with a significantly increased number of apoptotic cells predominantly being found in microinfarctions and mostly having cardiomyocytes morphology (Figure 2(b)). Manual count of TUNEL-positive nuclei showed significantly higher apoptosis in MT^−/−^-mice compared to WT after 3 days of I/R. (Figure 2(c)). The mRNA expression of related mediators revealed a comparable, nonsignificantly higher expression of caspase-8 in both genotypes after I/R (Figure 2(d)), while the caspase-3 was significantly downregulated in MT^−/−^-hearts after 1 day of I/R (Figure 2(e)). Interestingly, MT^−/−^-hearts presented also with a significant downregulation of antiapoptotic mediator B-cell lymphoma (Bcl)-2 at the same time (Figure 2(f)). These findings indicate a MT-related regulation of apoptosis mediators in the murine heart.

**Figure 3:**
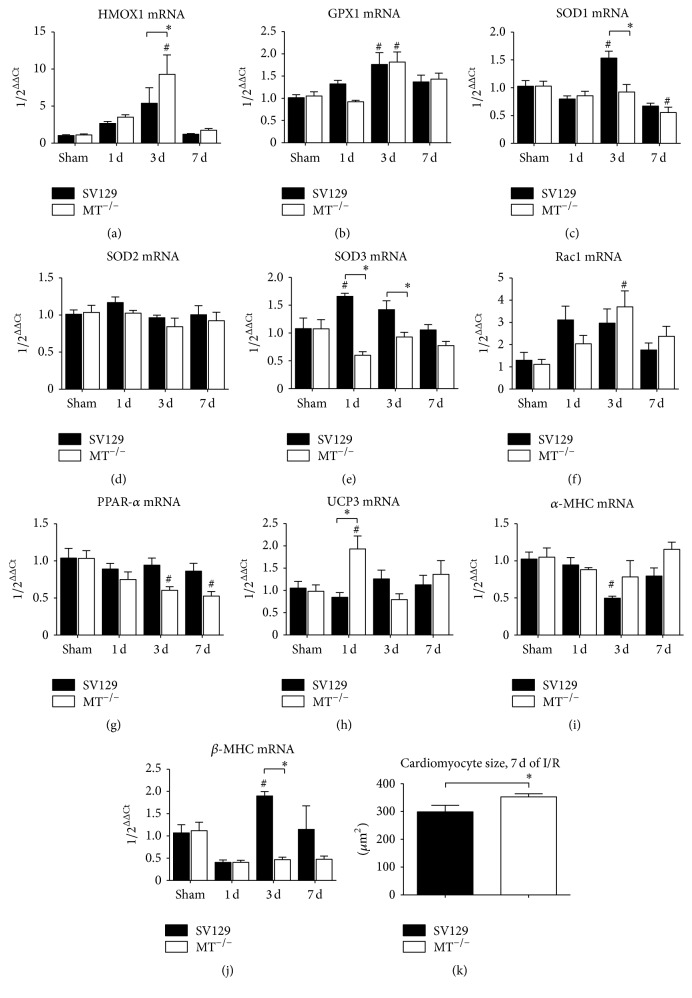
Impaired antioxidative control and maladaptation of contractile elements in MT^−/−^-hearts. RT-qPCR showing mRNA expression of (a) heme oxygenase (HMOX)1, (b) glutathione peroxidase (GPX)1, (c) superoxide dismutase (SOD)1, (d) SOD2, (e) SOD3, (f) Ras-related C3 botulinum toxin substrate (Rac)1, (g) peroxisome proliferator-activated receptor (PPAR)-*α*, (h) uncoupling protein (UCP) 3, (i) myosin heavy chain (MHC) isoform *α*, and (j) *β*-MHC isoform. (k) Quantification of cardiomyocyte size by area planimetry on collagen stained slides after 7 days of I/R. *n* = 8–10/group; RT-qPCR using Taqman, mRNA expression is related to shams and GAPDH using comparative ΔΔCt-method; *∗* indicates* P* ≤ 0.05 between the genotypes; # indicates* P* ≤ 0.05 versus respective sham.

Since I/R leads to an excessive reactive oxygen species production, which may cause apoptosis of cardiomyocytes, we investigated expression of antioxidative mediators. Heme oxygenase (HMOX)1 mRNA expression was significantly higher in MT^−/−^-mice when compared to WT after 3 days of I/R (Figure 3(a)). At the same time expression of glutathione peroxidase (GPX)1 was comparable between the genotypes (Figure 3(b)). The expression of superoxide dismutase (SOD) isoforms showed a significantly higher induction of SOD1 in WT-mice after 3 days of I/R (Figure 3(c)), no induction of SOD2 in both genotypes (Figure 3(d)), and significantly higher expression of SOD3 in WT-mice after 1 and 3 days of I/R (Figure 3(e)). In contrast, the expression of Ras-related C3 botulinum toxin substrate (Rac)1, the main regulator of NADPH oxidase, was significantly higher in MT^−/−^-mice after 3 days of I/R (Figure 3(f)), indicating an at least partially preserved potential to quench the reactive oxygen species. Additionally, MT^−/−^-mice presented with a significant downregulation of peroxisome proliferator-activated receptor (PPAR)-*α* after 3 and 7 days of I/R compared to sham (Figure 3(g)), indicating reduced utilization of fatty acids in order to further reduce oxidative stress burden [21]. Furthermore, the significant increase in uncoupling protein (UCP) 3 expression in MT^−/−^-mice after 1 day of I/R versus respective WT-mice (Figure 3(h)) supports this assumption [22]. Another important mechanism triggering apoptosis in cardiomyocytes represents the maladaptation of contractile elements, that is, myosin heavy chain (MHC), during repetitive ischemic episodes [5]. We found a significant downregulation of the less energetically efficient *α*-MHC isoform accompanied by an upregulation of the *β*-MHC in WT-mice, as expected during adaptation to I/R (Figures 3(i) and 3(j)). In contrast, MT^−/−^-mice presented with unchanged expression of the *α*-MHC accompanied by decreased expression of energetically more efficient *β*-MHC, a constellation indicative of a higher substrate consumption in their cardiomyocytes. Therefore, the increased loss of cardiomyocytes and apoptosis in MT^−/−^-hearts seems to be associated with impaired regulation of antioxidative enzymes and contractile elements. Subsequently, these mice compensate the loss of cardiomyocytes by an increased cardiomyocyte size and hypertrophy (Figure 3(k)).

### 3.3. Inflammatory Response in Ischemic MT^−/−^-Hearts

**Figure 4:**
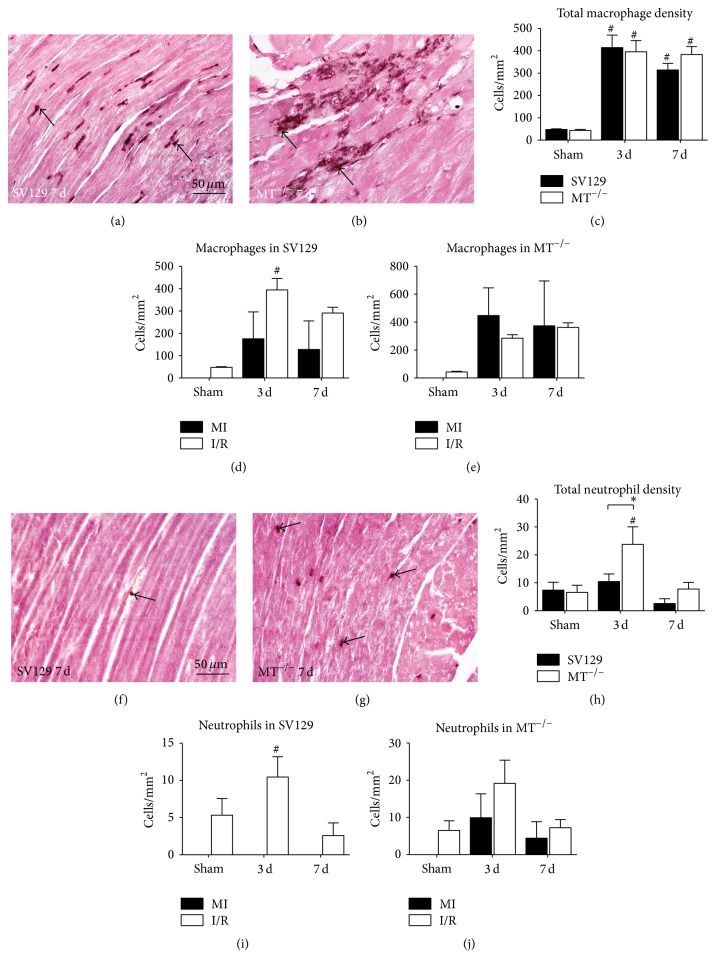
Macrophage and neutrophils infiltrate into areas of microinfarctions in MT^−/−^-hearts. MAC-2 staining of representative left ventricular sections after 7 days of (d) I/R demonstrates predominant interstitial macrophage infiltration (arrow) in WT-hearts (a), in contrast to increased macrophage density in microinfarctions (arrows) in MT^−/−^-hearts (b). (c) Quantification of MAC-2^+^ cells shows increased macrophage infiltration after 3 and 7 days of (d) I/R in both genotypes. Differential analysis of macrophage accumulation revealed that macrophages were mainly located in the interstitial spaces in WT-hearts (d) but to an equal number in microinfarctions in MT^−/−^-hearts (e). Representative MCA771G stained sections of WT- (f) and MT^−/−^-hearts (g) after 7 days of I/R (arrows: neutrophils). (h) Quantification of MCA771G^+^ cells showed higher neutrophil infiltration in MT^−/−^-hearts after 3 days of I/R compared to WT. Neutrophil distribution was comparable to macrophages (i and j). *n* = 8–10/group; scale bars in (a), (b), (f), and (g): 50 *μ*m; RT-qPCR using Taqman, mRNA expression is related to shams and GAPDH using comparative ΔΔCt-method; *∗* indicates* P* ≤ 0.05 between the genotypes; # indicates* P* ≤ 0.05 versus respective sham.

In order to investigate the potential of MT1 and MT2 to regulate inflammatory response we investigated migration of inflammatory cells into ischemic myocardium. Quantitative evaluation of MAC-2 positive macrophages showed a comparable cell density between the genotypes, which was significantly increased in both groups after 3 and 7 days when compared to their respective shams (Figures 4(a)–4(c)). Differential analysis of macrophage infiltration into ischemic area of WT-hearts revealed generally higher cell density in the interstitial space than in the few small areas of cardiomyocyte loss and being significantly different after 3 days of I/R (Figure 4(d)). In contrast, a comparable macrophage density was found between interstitial space and microinfarctions in MT^−/−^-mice during I/R (Figure 4(e)).

The analysis of MCA771G-positive neutrophils showed a significantly higher total cell density in MT^−/−^-mice after 3 days of I/R (Figures 4(f)–4(h)). The differential analysis revealed only scattered neutrophils in WT-myocardium being located in the interstitial space (Figure 4(i)). At the same time, MT^−/−^-mice presented with neutrophils located both in interstitial space and in microinfarctions (Figure 4(j)).

**Figure 5:**
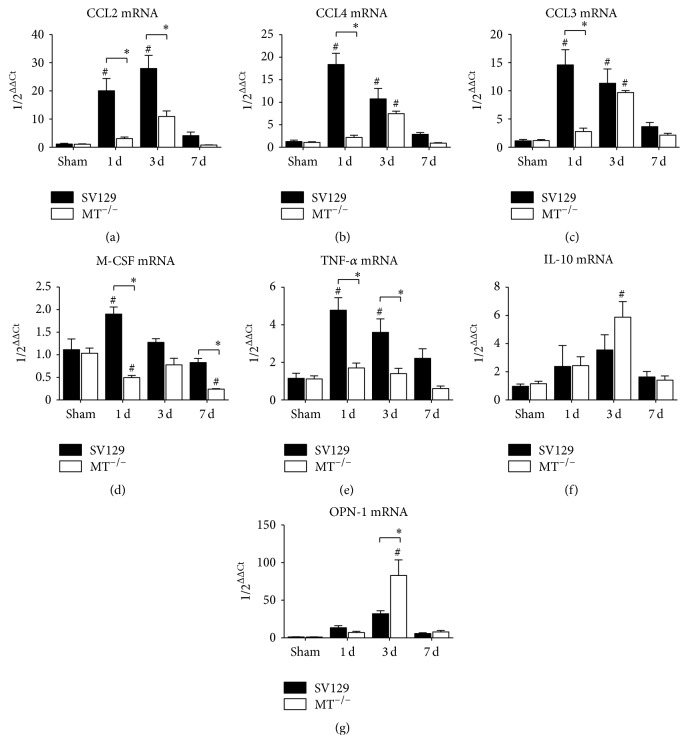
Gene expression of inflammatory mediators. Gene expression of macrophage-related chemokines (a) CCL2 and (b) CCL4 and (c) neutrophil-related CCL3 as well as cytokines (d) macrophage colony-stimulating factor (M-CSF), (e) TNF-*α*, (f) IL-10, and (g) osteopontin (OPN)-1. *n* = 8–10/group; RT-qPCR using Taqman, mRNA expression is related to shams and GAPDH using comparative ΔΔCt-method; *∗* indicates* P* ≤ 0.05 between the genotypes; # indicates* P* ≤ 0.05 versus respective sham.

In the next step we measured the mRNA expression of chemokines and cytokines related to the migration of inflammatory cells into ischemic myocardium. The expression of the most potent macrophage chemoattractant CCL2 was significantly higher in WT- than in MT^−/−^-hearts (Figure 5(a)). The chemokine CCL4 showed a similar expression pattern between the genotypes while having only one significant difference after 3 days of I/R (Figure 5(b)). Interestingly, at this time point MT^−/−^-mice presented with a significant induction of CCL4 when compared to their shams. In addition, the expression of the neutrophil-related chemokine CCL3 followed the same pattern as the monocyte/macrophage-related CCL4 by having a significantly higher expression in WT-hearts compared to MT^−/−^-hearts after 1 day of I/R, in contrast to a comparable induction after 3 days of I/R (Figure 5(c)). In contrast, the expression of the cytokine macrophage colony-stimulating factor (M-CSF) was again significantly higher in WT-mice, whereas MT^−/−^-mice presented even with a significant downregulation of it when compared to their shams (Figure 5(d)). The expression of the cytokine TNF-*α* was also significantly higher during I/R when compared to MT^−/−^-mice (Figure 5(e)). Still, the upregulation of the anti-inflammatory cytokine IL-10 was comparable between the groups, with only significant difference between 3 days of I/R and respective shams in MT^−/−^-mice (Figure 5(f)). The expression of macrophage maturation marker osteopontin- (OPN-) 1 was significantly increased after 3 days of I/R in MT^−/−^-mice (Figure 5(g)). Taken together, the MT^−/−^-mice had a generally lower expression of proinflammatory chemokines and cytokines during I/R but also a preserved capability for resolution of inflammatory response and timely onset of myocardial remodeling.

### 3.4. Repetitive I/R Leads to Adverse Myocardial Remodeling in MT^−/−^-Mice

**Figure 6:**
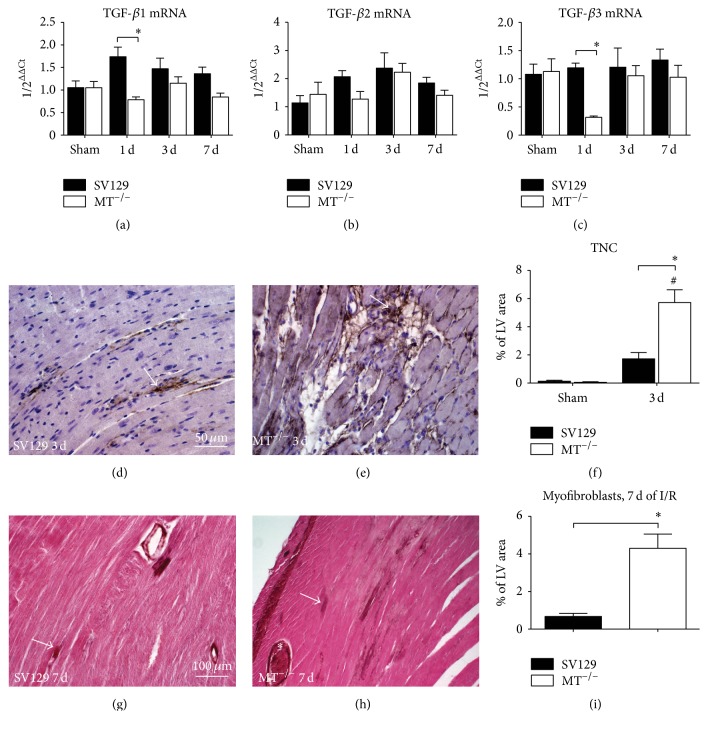
Adverse myocardial remodeling in MT^−/−^-mice. Gene expression of remodeling-related transforming growth factor *β* isoforms (a) TGF-*β*1, (b) -*β*2, and (c) -*β*3. Representative tenascin C (TNC) staining of WT- ((d), arrow) and MT^−/−^-hearts ((e), arrow) after 3 days of (d) I/R as well as the corresponding planimetric analysis (f). Representative left ventricular sections stained for myofibroblast marker *α*-smooth muscle actin (*α*-SMAC) after 7 days of I/R show (g) only few interstitial myofibroblasts in WT-hearts (arrow) but (h) strong myofibroblasts staining predominantly in microinfarctions (arrow) of MT^−/−^-hearts after 7 days of I/R. (i) Planimetric analysis of *α*-SMAC^+^ in both genotypes. *n* = 8–10/group; scale bars in (d) and (e): 50 *μ*m, in (g) and (h): 100 *μ*m; RT-qPCR using Taqman; mRNA expression is related to shams and GAPDH using comparative ΔΔCt-method; *∗* indicates* P* ≤ 0.05 between the genotypes; # indicates* P* ≤ 0.05 versus respective sham.

Timely onset and intensity of myocardial remodeling is important in cardioprotection in order to limit the expansion of myocardial injury. The mRNA expression of remodeling-related transforming growth factor- (TGF-) *β*1 isoform showed a higher level in WT-mice being only significant after 1 day of I/R when compared to the MT^−/−^-mice (Figure 6(a)). The *β*2 isoform of it showed no induction or difference between the genotypes (Figure 6(b)), while the *β*3 isoform presented with a significantly reduced expression after 1 day of I/R in MT^−/−^-mice compared to WT (Figure 6(c)). WT-mice presented with scattered tenascin C (TNC) staining throughout the interstitial space of the ischemic area (Figure 6(d)). In contrast, the MT^−/−^-mice revealed a significantly stronger staining signal (Figure 6(e)), as calculated upon its planimetric evaluation (Figure 6(f)). Similarly, WT-mice presented with a rather fine interstitial *α*-smooth muscle actin (*α*-SMAC) staining of myofibroblasts in contrast to a strong appearance of these cells in MT^−/−^-mice (Figures 6(g) and 6(h)). Planimetric analysis of this staining revealed a significantly larger percentage of the total left ventricular area in MT^−/−^-mice than in WT-mice (Figure 6(i)).

**Figure 7:**
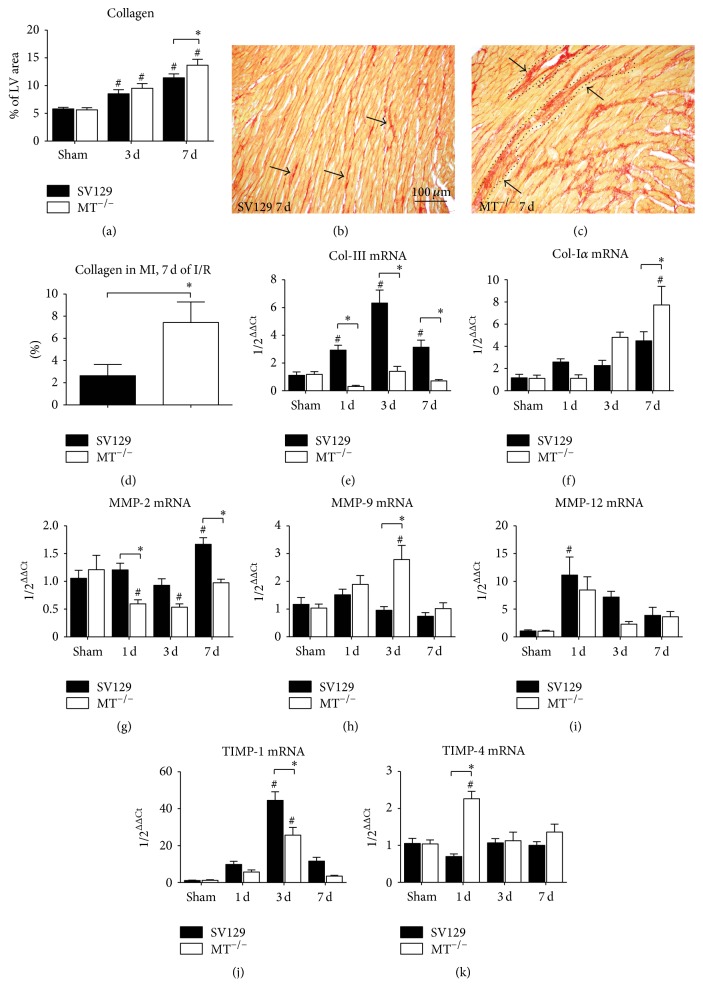
Scar formation and inadequate extracellular matrix turnover in MT^−/−^-mice. (a) Planimetric analysis of picrosirius red stainings shows increasing total collagen area after 3 and 7 days (d) in both genotypes. Representative picrosirius red stainings after 7 days of I/R shows (b) interstitial fibrosis (arrows) in WT-heart, in contrast to (c) dense collagen deposition in microinfarctions (arrows, dotted line: microinfarcted area) in MT^−/−^-hearts. (d) Significantly higher collagen deposition in microinfarcted areas in MT^−/−^-hearts after 7 days of I/R compared to WT. Evaluation of mRNA expression of (e) collagen (Col)-III, (f) Col-I*α*, (g) metalloproteinase (MMP)-2, (h) MMP-9, and (i) MMP-12 as well as (j) tissue inhibitor of MMP (TIMP)-1 and (k) TIMP-4. *n* = 8–10/group; scale bars in (b) and (c): 100 *μ*m; RT-qPCR using Taqman, mRNA expression is related to shams and GAPDH using comparative ΔΔCt-method; *∗* indicates* P* ≤ 0.05 between the genotypes; # indicates* P* ≤ 0.05 versus respective sham.

The higher intensity of myocardial remodeling seems to be attributable to the increased loss of cardiomyocytes and it resulted in significantly increased collagen area as a percentage of the total left ventricular area as well as microinfarctions in MT^−/−^-mice (Figures 7(a)–7(c)). We also performed a differential analysis of collagen deposition and found significantly higher percentage of it in microinfarctions of MT^−/−^-mice when compared to WT-mice (Figure 7(d)). The mRNA expression of the reversible deposited collagen (Col)-III revealed a significantly higher level in WT- than in MT^−/−^-mice, which practically showed downregulation of it (Figure 7(e)). As expected from histology we found significantly higher expression of the irreversibly deposited collagen (Col)-I*α* form in MT^−/−^-mice compared to WT (Figure 7(f)). Further analysis of matrix metalloproteinases (MMP) and their tissue inhibitors (TIMP) revealed a significantly higher expression of MMP-2 in WT-mice during I/R (Figure 7(g)). In contrast, the MT^−/−^-mice presented with a significantly higher induction of MMP-9 after 3 days of I/R (Figure 7(h)). The expression of MMP-12 was comparable between the genotypes and here WT-mice showed a significant induction after 1 day of I/R when compared to their shams (Figure 7(i)). The expression of TIMP-1 showed a significantly higher induction after 3 days I/R in WT- compared to MT^−/−^-mice, while both genotypes showed significant upregulation when compared to their respective shams at this time (Figure 7(j)). Interestingly, the MT^−/−^-mice presented with a significantly higher induction of TIMP-4 after 1 day I/R compared to WT and shams (Figure 7(k)). We also calculated the ratio of MMP-9 to TIMP-1 mRNA expression and found a significantly lower ratio of 0.02 in WT-hearts when compared to 0.11 in MT^−/−^-hearts after 3 days of I/R. The ratio of MMP-2 to TIMP-4 presented with a significantly higher ratio of 1.73 in WT-hearts when compared to 0.26 in MT^−/−^-hearts after 1 day of I/R. Taken together, MT^−/−^-mice experienced an intense myocardial remodeling with more collagen deposition and imbalanced expression of MMP and TIMP in the ischemic area when compared to WT-mice.

## 4. Discussion

Previous studies utilized cardiac-specific overexpression of metallothionein to introduce its involvement in cardioprotective mechanisms [23]. One of them utilized an* ex vivo* model of reperfused infarction to show a reduced infarction size [9]. Another study used an* in vivo* model of coronary occlusion followed by four hours reperfusion and suggested that the protection of metallothionein excess against cardiomyocyte apoptosis is mediated via reduction of reactive oxygen species [10]. We have chosen a different approach to characterize the role of metallothionein in myocardial ischemia by using MT^−/−^-mice in a model of brief repetitive I/R without myocardial infarction. This nonlethal ischemia model is of clinical relevance in regard to prevention and early therapy before myocardial injury occurs [2]. Our data provide not only further evidence for cardioprotective effects of metallothionein but also describe downstream effects on mediators during adaptation, inflammatory response, and myocardial remodeling in ischemic, noninfarcted cardiomyopathy. Furthermore, we provide evidence that the wild type SV129 mouse strain shares the same pattern of functional, morphological, and molecular events after repetitive I/R as the previously published C57/Bl6 strain [3, 5].

The murine model of brief repetitive I/R leads to a transient inflammatory reaction involving predominately chemokine induction and macrophage migration into ischemic myocardium [3]. This results in an interstitial fibrosis and left ventricular dysfunction, which are both reversible after discontinuation of the I/R protocol. Our current study provides detailed characterization of these events in SV129 mouse strain for the first time. We could show a very much comparable course of chemokine and cytokine induction, macrophage migration, and remodeling-related factors in comparison to the C57/Bl6 mouse strain. Also, the extent of interstitial fibrosis and left ventricular dysfunction are at the same level in both wild type mouse strains. This is important not only for interpretation of the MT^−/−^-mice data but also for future studies using this model in other genetically manipulated mice.

The MT^−/−^-mice experienced an irreversible loss of cardiomyocytes already after 3 days of I/R and this pathology became clearly delineated after 7 days of I/R. Even though we found no significant difference in regional pump function of the anterior left ventricular wall between the genotypes after 7 days of I/R, there was a significantly worse global left ventricular function in MT^−/−^-mice at the same time. Our data provide evidence for cardiomyocyte loss—at least in part by apoptosis—in the lack of MT1 and MT2 and thereby confirm the previously published association with apoptosis [10, 24]. In addition, our data on apoptosis related mediators suggest a link between expression of metallothionein and the antiapoptotic Bcl2 in prevention of cardiomyocyte apoptosis. This is in concordance with the reported role of metallothionein in Akt-pathway as cardioprotective mechanism [13]. Also, our data support the previously published association of metallothionein with antioxidative enzymes in myocardial injury [10]. Our findings indicate a decreased induction of antioxidative enzymes in MT^−/−^-mice but a preserved activation of Rac1 and thereby induction of NADPH oxidase. Since activation of Rac1 has been associated with Notch2 regulation for neuroprotection after I/R injury [25], we assume the contribution of this effect to prevent even a higher loss of cardiomyocytes in MT^−/−^-mice. Another effect aiming at further reduction of reactive oxygen species production is indicated by downregulation of PPAR-*α* in MT^−/−^-mice, resulting in decreased utilization of fatty acids [19, 21]. This is further supported by upregulation of UCP3 in MT^−/−^-mice, which represents an additional mechanism to counteract the high burden of free radicals [22]. Therefore, the published data and our findings strongly indicate a redundancy in these mechanisms in order to prevent detrimental loss of cardiac function. While other studies provided evidence for the association of metallothionein with STAT-3 [12] and Akt-pathway [13] during cardioprotection, we investigated the contractile elements of cardiomyocytes. MT^−/−^-mice presented with malfunction in adaptation of *α*- and *β*-MHC resulting in high substrate consumption, which is also contributing to increased cardiomyocyte apoptosis [5, 21]. In consequence, the remaining viable cardiomyocytes presented with hypertrophic response in MT^−/−^-mice and one can therefore speculate that the continuation of repetitive I/R beyond 7 days would cause further loss of cardiomyocytes.

The production of reactive oxygen species leads to inflammatory reaction during repetitive I/R. Our data show a clear dependence of initiation of chemokine induction on the presence of metallothionein in this model. This may be specific to the low level, nonbacterial inflammation in noninfarcted heart, since a study reported that* Helicobacter pylori* infected MT-null mice (C57/Bl6 background) have a significantly higher induction of CCL2 [26]. The chemokines took the lead in MT^−/−^-hearts to initiate an inflammatory reaction after 3 days of I/R and attract macrophages and neutrophils, whereas the cytokine expression was even downregulated. The higher neutrophils density in MT^−/−^-hearts is clearly associated with the cardiomyocyte apoptosis and the need for debris removal in these hearts when compared to the very faint loss of cardiomyocytes in WT-mice without microinfarctions. The higher migration of inflammatory cells into ischemic hearts of MT^−/−^-mice is still associated with a timely resolution of inflammatory response, as shown by the induction of anti-inflammatory cytokine IL-10 and a macrophage maturation marker OPN-1 [27]. This suggests a rapid transition to myocardial remodeling in order to preserve myocardial function [1].

Interstitial fibrosis represents the hallmark of myocardial remodeling during repetitive I/R. Our findings showed the lack of induction or even downregulation of the expression of two transforming growth factor *β* isoforms TGF-*β*1 and -*β*3 after 1 day of I/R, which may be associated with the initiation of a strong remodeling response. Our data revealed first evidence for metallothionein-related deposition of TNC in ischemic murine hearts. Since TNC is related to early stages of tissue remodeling and embryonic development [28], its stronger production in MT^−/−^-mice can be associated with significantly increased differentiation of myofibroblasts in their hearts. This results in a higher intensity of myocardial remodeling due to the lack of metallothionein, which is in accordance with the previously published data on attenuated myocardial remodeling in MT-overexpressing mice [11]. Therefore, metallothionein seems also to be directly involved in modulation of myocardial remodeling, but we can only speculate on related mechanisms. The analysis of collagen deposition and production revealed practically a lack of the reversible collagen III induction in MT^−/−^-mice. This seems be associated with the increased need for deposition of stable collagen I*α* in the scars of microinfarctions in these mice, as further indicated by its strong mRNA induction. The data on MMP- and TIMP-expression also support our interpretation of formation of nonreversible collagen deposition during scar formation in MT^−/−^-mice. The MMP/TIMP ratio also indicates a shift in balance between production of extracellular matrix components and adverse remodeling in MT^−/−^-hearts.

We did not investigate the reversibility of the phenotype in MT^−/−^-mice since our previous studies showed permanently impaired function and scar persistence 60 days after discontinuation of the I/R in mice with microinfarctions [5]. Also, the interpretation of our findings may have a weakness which is that it is only based on mRNA expression without protein data. Still, the latter is relative since most of the mediators we investigated are transcriptionally well regulated.

## 5. Conclusions

In conclusion, metallothionein seems to provide cardioprotection via modulation of antioxidative enzymes and contractile elements, regulation of inflammatory response, and subsequent myocardial remodeling in murine model of ischemic cardiomyopathy without infarction. Our findings may open a therapeutic perspective for targeting metallothionein in prevention or treatment of ischemic heart disease.
